# The clinical implications of fasting serum insulin levels in patients with insulin-treated type 2 diabetes: a cross-sectional survey

**DOI:** 10.3389/fcdhc.2023.1172208

**Published:** 2023-05-30

**Authors:** Lingli Zhou, Yingying Luo, Yan Wang, Yao Cheng, Rui Zhang, Simin Zhang, Siqian Gong, Xueyao Han, Linong Ji

**Affiliations:** ^1^Department of Endocrinology and Metabolism, Peking University People’s Hospital, Beijing, China; ^2^Department of Endocrinology and Metabolism, People’s Hospital of Deyang City, Deyang, Sichuan, China

**Keywords:** fasting insulin levels, insulin resistance, insulin antibodies, type 2 diabetes, hyperinsulinemia, insulin treatment

## Abstract

**Objective:**

This study aimed to investigate the clinical implications of fasting serum insulin (FINS) levels in subjects with type 2 diabetes who were receiving insulin therapy.

**Methods:**

A total of 1,553 subjects with type 2 diabetes [774 subjects who had never received insulin treatment (N-INS) and 779 subjects who were receiving insulin therapy (constant insulin treatment, C-INS)] admitted to the Department of Endocrinology and Metabolism of Peking University People’s Hospital were enrolled in this study. Their FINS levels were measured and those with hyperinsulinemia were identified. The underlying mechanisms of hyperinsulinemia were revealed by measuring insulin antibodies (IAs) and analyzing changes in FINS levels before and after polyethylene glycol (PEG) precipitation. In addition, the clinical characteristics of patients with different types of hyperinsulinemia were compared.

**Results:**

Higher FINS levels and a higher incidence (43.8%, 341/779) of hyperinsulinemia (FINS > 15μIU/mL) were observed in subjects with C-INS than in subjects with N-INS. Among subjects with C-INS and hyperinsulinemia, 66.9% (228/341) were IAs positive, and the incidence of IAs was found to be positively associated with FINS level. By performing PEG precipitation, we found that all subjects without IAs (i.e., those with real hyperinsulinemia) and 31.1% of subjects (71/228) with IAs (i.e., those with both real and IAs-related hyperinsulinemia) still had hyperinsulinemia after PEG precipitation, whereas FINS levels in the other 68.9% of subjects (157/228) with IAs were normal (IAs-related hyperinsulinemia) after PEG precipitation. Comparisons between the groups showed that subjects with real hyperinsulinemia showed more obvious insulin resistance characteristics, including higher lipid levels, BMIs, and homoeostasis model assessment2-estimated insulin resistance (HOMA2-IR) index, and were more likely to have hypertension, obesity, and metabolic syndromes (*p* < 0.05). However, the risk of hypoglycemia and glucose variability increased significantly in subjects with IAs compared with those without IAs. A cutoff of FINS to serum C-peptide ratio (≥ 9.3μIU/ng) could be used to screen IAs in clinical practice with 83.3% sensitivity and 70% specificity.

**Conclusions:**

It is necessary to measure FINS in subjects with C-INS to distinguish between types of hyperinsulinemia, which should help to tailor treatment regimens.

## 1 Introduction

Type 2 diabetes is characterized by progressive hyperglycemia due to impaired insulin secretion or/and insulin resistance. Hyperinsulinemia is an important indicator of insulin resistance in patients with type 2 diabetes. In clinical practice, insulin therapy is now widely used in the management of type 2 diabetes worldwide; however, it can cause iatrogenic hyperinsulinemia. According to the available guidelines, insulin or insulin analogs should be administered when the target blood glucose level in patients with type 2 diabetes is not achieved. Although some patients have hyperinsulinemia or insulin resistance, they are treated with insulin or insulin analogs when treatment with adequate doses of other available anti-diabetic drugs does not lead to the target optimized blood glucose control. However, intensive insulin treatment has been shown to increase the risk of negative cardiovascular outcomes (1–3), which suggests that hyperinsulinemia due to the over-administration of insulin or insulin analogs can lead to weight gain and hypoglycemia, both of which are potentially harmful to patient health and should therefore be prevented as much as possible.

Conversely, exogenous insulin can induce autoimmune syndrome (EIAS), which mainly manifests as hyperinsulinemia and unexpected hypoglycemia (4). Thus, hyperinsulinemia in insulin- or insulin analog-treated patients with type 2 diabetes can be categorized as either immunity-induced or non-immunity-induced hyperinsulinemia (real hyperinsulinemia). Measuring fasting serum insulin (FINS) levels and differentiating between the two types of hyperinsulinemia might provide us with useful information to tailor the treatment regimens of patients receiving either insulin or insulin analog treatment.

In fact, in contrast to serum C-peptide levels, FINS levels were seldom measured, and doing so is usually considered unnecessary for insulin- or insulin analog-treated patients with type 2 diabetes, because serum C-peptide levels reflect endogenous insulin secretion, whereas FINS levels include both endogenous and exogenous insulin. Thus, it is not surprising that little was known about the prevalence of hyperinsulinemia and its potential clinical value in patients with type 2 diabetes who were treated with insulin or insulin analogs.

Therefore, the aims of this study were to investigate (1) the prevalence of hyperinsulinemia (2), the differences between the clinical characteristics of patients with immunity-induced and real hyperinsulinemia, and (3) the clinical implications of FINS levels in patients with type 2 diabetes who were treated with insulin or insulin analogs.

## 2 Methods

### 2.1 Diagnostic criteria for diabetes, obesity, and metabolic syndrome

Diabetes was clinically diagnosed according to 1999 WHO criteria. We used the Chinese-specific diagnostic criteria to define obesity (i.e., a BMI of ≥ 28 kg/m^2^) (5). Metabolic syndrome (MS) was diagnosed using the International Diabetes Federation consensus worldwide definition (6).

### 22 Definition of hyperinsulinemia

As described in our recent study (7), the FINS levels of 150 healthy subjects with normal glucose tolerance and normal components of MS were measured. The 97.5th percentile of FINS levels was used to determine the cutoff value of FINS for identifying hyperinsulinemia (FINS > 15μIU/mL).

### 2.3 Study subjects

**Figure 1:**
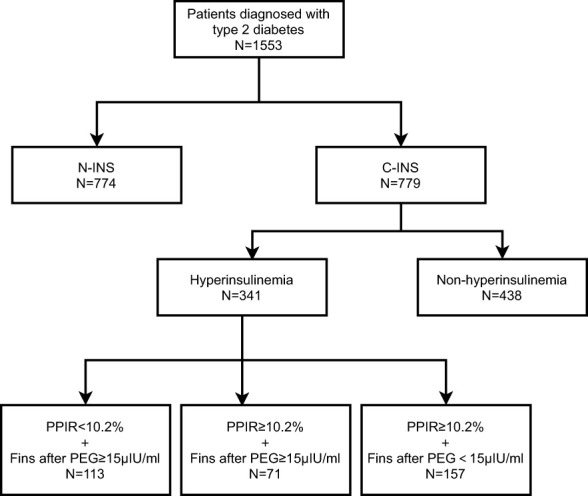
Flowchart of patients included in study.

For patient population 1, we recruited 1,553 patients with type 2 diabetes who had been hospitalized at the Department of Endocrinology and Metabolism of Peking University People’s Hospital because of poor glucose control from December 2015 to October 2020. Of those, 774 subjects had been treated with non-insulin hypoglycemic drugs and had never received insulin treatment (non-insulin-treated type 2 diabetes, N-INS), and 779 subjects had been receiving insulin therapy for at least 1 month (constant insulin treatment, C-INS) (**Figure 1**). The inclusion criteria were men or women who were aged 18–80 years, had been diagnosed with type 2 diabetes, and had received non-insulin drug or insulin treatments. Subjects with one or more of the following conditions were excluded: (1) pregnancy; (2) a medical history of autoimmune diseases, such as rheumatoid arthritis, Sjögren’s syndrome, systemic lupus erythematosus, and Graves’ disease; (3) type 1 diabetes; (4) positive glutamate decarboxylase antibody (GADA) or islet cell antibody (ICA) test results; (5) specific types of diabetes; and (6) an estimated glomerular filtration rate (eGFR) of < 60mL/min/1.73m^2^ (avoiding the possible effects of poor renal insulin clearance). Their clinical characteristics are depicted in **Table 1**.

**Table 1:** Baseline clinical and biochemical characteristics of subjects with type 2 diabetes with non-insulin and insulin treatment.

	All subjects	N-INS	C-INS	*P*-value
Subjects (*n*)	1553	774	779	
Ages (years)	57 ± 12	54 ± 13	59 ± 11	< 0.001
Age at diagnosis (years)	45 ± 11	46 ± 12	43 ± 11	< 0.001
Sex, male (*n*,%)	937 (60.3%)	500 (64.6%)	437 (56.1%)	0.001
Duration of diabetes (years)	12.0 (5.0, 19.0)	7.0 (2.0, 12.3)	16.0 (10.0, 20.0)	< 0.001
BMI (kg/m^2^)	27.3 ± 3.7	27.4 ± 3.8	27.2 ± 3.6	0.194
SBP (mmHg)	136 ± 19	136 ± 18	135 ± 19	0.104
DBP (mmHg)	77 ± 12	79 ± 12	75 ± 11	< 0.001
Waist circumferences (Male, cm)	98.2 ± 8.9	97.9 ± 9.1	98.6 ± 8.6	0.305
Waist circumferences (Female, cm)	94.9 ± 10.1	94.2 ± 9.8	95.4 ± 10.3	0.301
LDL-C (mmol/L)	2.64 ± 0.87	2.73 ± 0.88	2.55 ± 0.85	< 0.001
TC (mmol/L)	4.28 ± 1.46	4.43 ± 1.71	4.14 ± 1.15	< 0.001
TG (mmol/L)	1.67 (1.20, 2.36)	1.68 (1.19, 2.46)	1.66 (1.21, 2.32)	0.392
HDL-C (Male, mmol/l)	0.96 ± 0.30	0.96 ± 0.30	0.96 ± 0.30	0.521
HDL-C (female, mmol/L)	1.08 ± 0.25	1.08 ± 0.22	1.09 ± 0.28	0.675
FBG (mmol/L)	7.71 ± 2.85	7.66 ± 2.84	7.76 ± 2.87	0.337
HbA1c (%)	9.2 ± 2.1	9.3 ± 2.4	9.1 ± 1.7	0.020
C-peptide (ng/mL)	2.03 (1.48, 2.81)	2.34 (1.75, 3.09)	1.78 (1.29, 2.46)	< 0.001
FINS before PEG (μIU/mL)	11.7 (8.1, 17.2)	10.2 (7.6, 14.0)	13.6 (8.7, 21.9)	< 0.001
FINS after PEG (μIU/mL)	10.8 (7.8, 15.0)	11.2 (8.3, 14.8)	10.5 (7.4, 15.1)	0.028
PPIR (%)	–3.5 (–9.7, 8.7)	–6.8(–12.3, -2.3)	4.5 (–5.6, 44.0)	< 0.001
Hyperinsulinemia before PEG (*n*, %)	506 (32.6%)	165 (21.3%)	341 (43.8%)	< 0.001
Hyperinsulinemia after PEG (*n*, %)	383 (24.7%)	185 (23.9%)	198 (26.4%)	0.351
IA positive (ELISA, n/*n*,%)	79/1460, 5.1%	3/709, 0.4%	76/751, 10.1%	< 0.001
Diabetic retinopathy (n/*n*,%)	418/1532, 26.9%	141/771, 18.3%	277/761, 36.4%	< 0.001
UACR (mg/g)	8.4 (4.4, 26.0)	7.44 (4.1, 17.7)	10.0 (4.7, 45.7)	< 0.001

BMI, body mass index; SBP, systolic blood pressure; DBP, diastolic blood pressure; LDL-C, low density lipoprotein-cholesterol; TC, total cholesterol; HDL-C, high density lipoprotein-cholesterol; FBG, fasting blood glucose; HbA1c, glycosylated hemoglobin A1c; FINS before PEG, fasting insulin concentration before PEG-precipitation; FINS after PEG, fasting insulin concentration after PEG-precipitation; PPIR, PEG-precipitated insulin ratio; IA, insulin autoimmune antibody; UACR, urinary albumin creatinine rate.

Patient population 2 comprised 120 patients who were randomly selected from patient population 1 who had been receiving insulin treatment. After written informed consent was obtained, serum samples were collected and used to evaluate the performances of several assays for detecting insulin antibodies (IAs).

Patient population 3 comprised 44 patients selected from patient population 1 to explore the effects of IAs-related hyperinsulinemia on glucose variability by flash glucose monitoring (FGM) after they provided written informed consent, of whom 22 were patients with positive IAs results and 22 were patients with negative IAs results. Matching was performed based on demographic characteristics [age, gender, and hemoglobin A1c (HbA1c) level].

The study protocol complies with the principles of the Declaration of Helsinki and was approved by the Ethics Committee of Peking University People’s Hospital. The individuals in patient population 2 and patient population 3 provided written informed consent and the other patients’ consent was waived for this retrospective analysis.

### 2.4 Medical histories, physical examination, and laboratory measurements

Medical histories, including demographic information, details of glucose-lowering therapies, history of hypoglycemia, and comorbidities, were collected by physicians. Hypertension was defined as a systolic pressure of ≥ 140 mmHg and/or a diastolic pressure of ≥ 90 mmHg and/or the use of antihypertensive drugs. BMI was calculated as weight (kg) divided by the square of height (m). All blood samples were obtained while patients were in a fasting state in the morning. HbA1c levels were measured using high-performance liquid chromatography (Premier Hb9210, USA). Serum C-peptide was tested by ELISA (Cobas e601, Roche Diagnostics, Germany). Serum total cholesterol (TC), triglycerides (TG), low-density lipoprotein cholesterol (LDL-C), high-density lipoprotein cholesterol (HDL-C), uric acid (UA), and serum creatinine (CRE) levels were determined using enzymatic methods on an automatic biochemical analyzer (Hitachi 7170 A, Tokyo, Japan). Urinary albumin/creatinine ratio (UACR, mg/g) was measured on a COBAS Integra 400 Plus System (Roche Diagnostics, Basel, Switzerland). The mean UACR was calculated based on two or three independent measurements. The presence of IAs was detected using an ELISA kit (ORGENTEC Diagnostika GmbH, Germany) for all subjects and by radioimmunoassay (RIA) (RiaRSR™, United Kingdom) and another ELISA kit (Biomerical, USA) for subjects in patient population 2. Retinopathy was assessed using fundus photography (TRC.NW400, Topcon Inc., Japan). Homoeostasis model assessment2-estimated insulin resistance (HOMA2-IR) was calculated using the formula available at http://www.dtu.ox.ac.uk/homacalculator/index. The eGFR was calculated using the Chronic Kidney Disease Epidemiology Collaboration (CKD-EPI) formula.

### 2.5 Determining IAs through polyethylene glycol precipitation

Polyethylene glycol (PEG) precipitation was carried out as previously described (8). In brief, 100μL of serum was added to the same volume of 25% cold PEG 6000 (Sinopharm chemical reagent Beijing Co., Ltd. #20161104) and mixed vigorously, followed by centrifugation at 3,000×g for 30 min. Both serum insulin levels (FINS before PEG, also called direct FINS) and the insulin levels in the supernatant (FINS after PEG, also called free FINS) after PEG precipitation were measured on an electrochemiluminescence immunoassay analyzer (Cobas e601, Roche Diagnostic, Germany). As the manufacturer had indicated, the FINS measured by this kit showed no cross-reactivity with insulin aspart, insulin lispro, and insulin glargine (9, 10). The PEG/precipitated insulin ratio (PPIR), which represents the proportion of antibody-bound insulin, was calculated as (FINS before PEG – FINS after PEG)/FINS before PEG. Due to dilution bias during sample processing, the final result for subjects without IAs is often negative. The inter-assay and within-assay coefficient of variations (CVs) for the FINS after PEG precipitation were 2.79% and 2.16%, respectively.

### 2.6 FGM

The FreeStyle Libre system (Abbott Diabetes Care, Witney, Oxon, UK) with a sensor filament in the subcutaneous tissue that measures interstitial fluid glucose levels was used for continuous glucose monitoring for 2 weeks. The data were uploaded to a database when connected to the reporting software, and the glycemic variability parameters were calculated automatically.

### 2.7 Data analyses

Continuous variables were expressed as means ± SD. Since TG, UACR, C-peptide, and FINS were not normally distributed, these measurements were log-transformed (ln) and presented as medians and interquartile ranges. Between-group and multiple-group differences for normally distributed continuous parameters were determined using an independent *t*-test and one-way ANOVA, respectively. A chi-squared test was performed to analyze the categorical variables. A *p*-value of < 0.05 was considered statistically significant for all comparisons. For determining the diagnostic sensitivity and specificity of clinical characteristics markers in predicting IAs, the receiver operating characteristic (ROC) curve was drawn, and the optimal cutoff value was identified by calculating Youden’s index. Statistical analyses were performed using SPSS 22.0 (IBM, Inc., New York, USA). The comparison of areas under the curve (AUC) was conducted using MedCalc software V20.106 (https://www.medcalc.org/).

## 3 Results

### 3.1 The performance of two commercial IAs detection kits and the prediction of IAs by calculating PPIR

**Figure 2:**
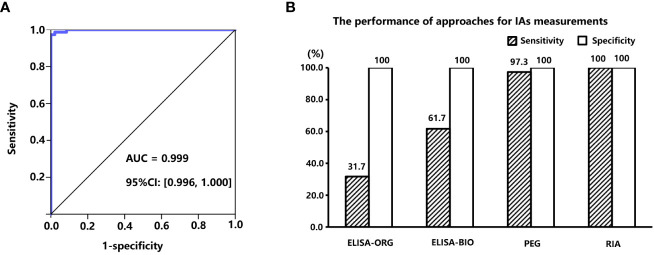
**(A)**, area under the receiver operation characteristic (ROC) curve of PPIR in diagnosing IAs. **(B)**, Comparisons of sensitivities and specificities between three different approaches for determining IAs by using RIA (RiaRSRTM, Avenue Park United Kingdom, United Kingdom) method as the “gold standard”, including two commercial ELISA kits manufactured by ORGENTEC Diagnostika GmbH, Mainz, Germany and Biomerical, USA, and polyethylene glycol (PEG) precipitation, respectively. IAs, insulin antibodies; PPIR, PEG-precipitated insulin ratio; RIA, radioimmunoassay.

To determine which method was the most appropriate to detect IAs, the performance of commercial ELISA IAs kits and calculated PPIR value through PEG precipitation was evaluated. A total of 120 serum samples from subjects with C-INS were tested using RIA (considered the gold standard), and 73 samples were positive for IAs (RIA-IAs). Of these, only 20 (27.4%) and 40 (54.8%) samples were also positive for IAs by using ELISA kit (ORGENTEC Diagnostika GmbH, Germany) and ELISA kit (Biomerical, USA), respectively. The PPIR values in subjects with positive RIA-IAs were significantly higher than in those with negative RIA-IAs [55.1% (25.2%, 73.4%) *vs*. –10.0% (–11.7%, –9.3%), *p* < 0.001]. The AUC of the PPIR value was 0.999 (95% CI: 0.996 to 1.000) and the optimal cutoff PPIR value for identifying positive RIA-IAs was 10.2%, with a sensitivity of 97.3% and a specificity of 100% (**Figure 2A**). The sensitivity of PPIR-predicted IAs (≥ 10.2%) was much higher than that of both commercial ELISA kits detected IAs and similar to RIA-IAs (**Figure 2B**). Therefore, IAs were considered positive when the PPIR value reached or exceeded 10.2% in our study.

### 3.2 The clinical characteristics of type 2 diabetic patients with and without insulin agents use

As shown in **Table 1**, compared with subjects with N-INS, subjects with C-INS, as we expected, were older, had been of a younger age when diagnosed with diabetes, had had diabetes for a longer duration, and had lower serum C-peptide levels and a higher prevalence of microvascular complications, although BMIs, FBGs, waist circumferences, TG, and HDL-C levels were similar between the groups. Higher direct FINS levels [13.6 (8.7, 21.8) *vs*. 10.2 (7.6, 14.0) μIU/mL, *p* < 0.001)] and a higher prevalence of hyperinsulinemia (43.8% *vs*. 21.3%, *p *< 0.001) were observed in subjects with C-INS. Free FINS levels were lower in subjects with C-INS than in those with N-INS [10.5 (7.4, 15.1) *vs*. 11.2 (8.3, 14.8) μIU/mL, *p* = 0.028].

The median PPIR value in subjects with C-INS was 4.5% (–5.6%, 44.0%), which was higher than in subjects with N-INS [6.8% (–12.3%, –2.3%), *p* < 0.001]. The 97.5th percentile of PPIR value in subjects with N-INS was 9.9%, which is very close to the PPIR cutoff value (10.2%). Out of 779 subjects, there were 325 (41.7%) and 341 (43.8%) subjects with C-INS with a PPIR value ≥ 9.9% and a PPIR value ≥ 10.2%, respectively; these values which were much higher than the percentage (10.1%) of IAs detected by the ELISA kit (ORGENTEC Diagnostika GmbH, Germany) (*p* < 0.001), also suggesting that compared to ELISA kits, PPIR-predicted IAs is more sensitive to detect IAs.

### 3.3 The distribution of IAs in insulin-treated type 2 diabetic patients with different FINS levels

**Figure 3:**
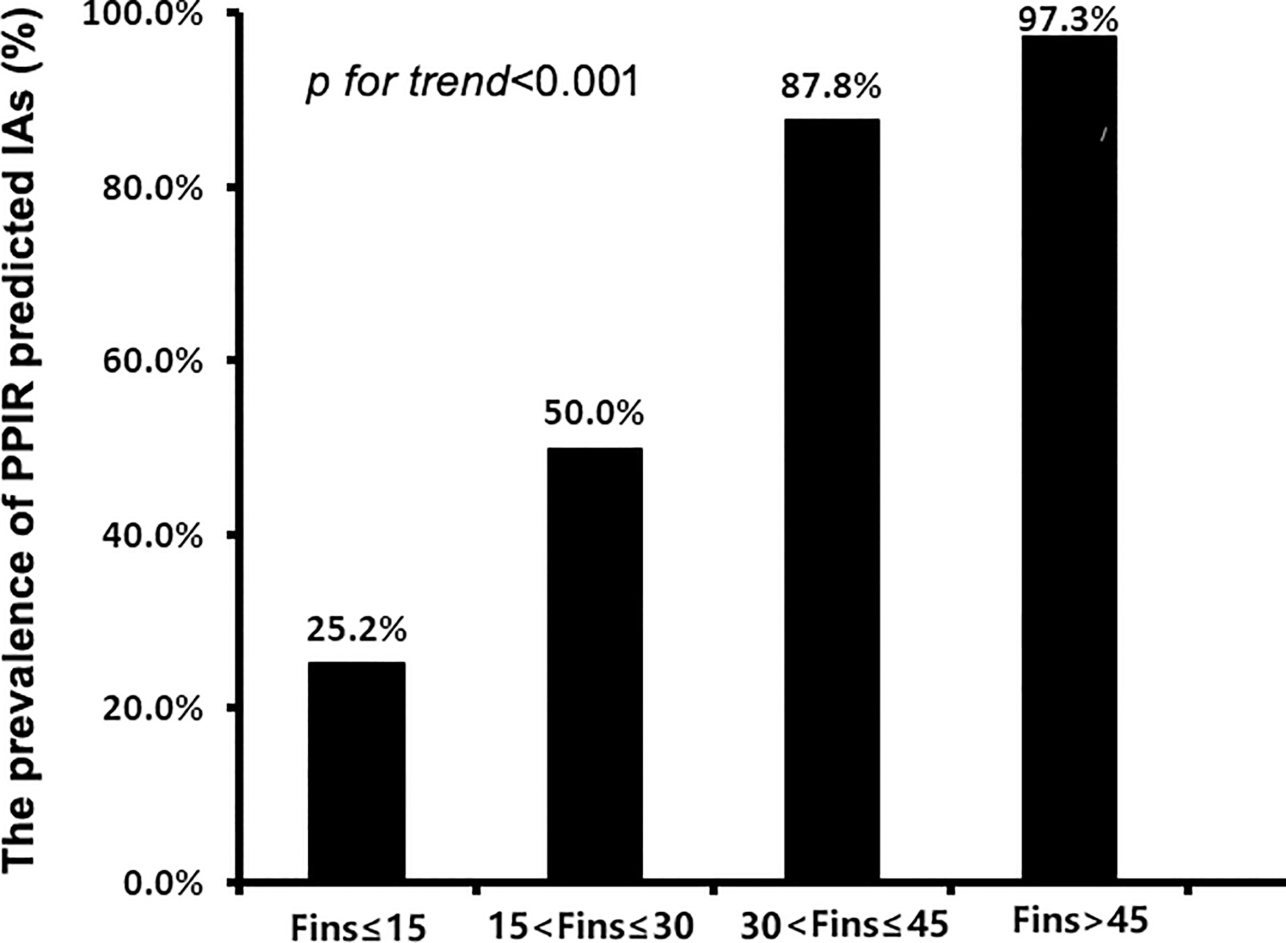
The prevalence of IAs in patients with different levels of fasting insulin concentrations (FINS). IAs, insulin antibodies.

To further investigate the relationships between FINS levels and IAs in insulin-treated type 2 diabetic subjects, we divided 779 subjects with C-INS into four groups according to their direct FINS levels: group 1, ≤ 15μIU/mL; group 2, 15–30μIU/mL; group 3, 30–45μIU/mL; and group 4, > 45μIU/mL. The IAs prevalence was 25.2%, 50.0%, 87.8%, and 97.3% in groups 1, 2, 3, and 4, respectively. With the increase in direct FINS levels, the prevalence of IAs also increased significantly (*p* for trend < 0.001) (**Figure 3**).

### 3.4 The clinical characteristics of patients with different mechanisms of hyperinsulinemia

As shown in **Table 1**, 341 out of 779 subjects with C-INS presented with hyperinsulinemia. Among them, 228 subjects (66.9%) showed positive IAs and 113 subjects (33.1%) had negative IAs. After PEG precipitation, all 113 subjects without IAs still showed hyperinsulinemia, as we expected (PPIR < 10.2% + FINS after PEG ≥ 15μIU/mL, real hyperinsulinemia). In contrast, in the 228 subjects with IAs, the free FINS levels for 157 subjects (68.9%) fell below 15μIU/mL (PPIR ≥ 10.2% + FINS after PEG < 15μIU/mL, IAs-related hyperinsulinemia), and the other 71 subjects (31.1%) still showed hyperinsulinemia, but to a lower extent (PPIR ≥ 10.2%+FINS after PEG ≥ 15μIU/mL, both real and IAs-related hyperinsulinemia). Subjects with real hyperinsulinemia were the youngest (*p* < 0.001), had developed diabetes at the youngest ages (*p* = 0.005) and for the shortest duration (*p* < 0.001), had the highest BMIs (*p* < 0.001), the highest serum C-peptide levels (*p* < 0.001), the greatest extents of insulin resistance assessed by HOMA2-IR (*p* < 0.001), the highest waist circumferences (*p* = 0.015 for male and *p* < 0.001 for female), and were mostly likely to be dyslipidemic (*p* < 0.001), obese (*p* < 0.001), and to develop MS (*p* = 0.001). The subjects with IAs-related hyperinsulinemia had the lowest serum C-peptide levels (*p* < 0.001), the worst beta-cell function as assessed by HOMA2-B (*p* = 0.003), and the highest prevalence of hypoglycemia (*p* = 0.028). There were no statistical differences in terms of FBG, HbA1c, and UACR levels and in the prevalences of hypertension and diabetic retinopathy among the three groups (**Table 2**).

**Table 2:** Comparisons of clinical characteristics between insulin-treated type 2 subjects with hyperinsulinemia with and without IAs.

PPIR < 10.2% FINS after PEG > 15μIU/mL (Real hyperinsulinemia)		PPIR ≥ 10.2%		*p*-value
		FINS after PEG >15μIU/mL (Real+IAs-related hyperinsulinemia)	FINS after PEG ≤15 μIU/mL (IAs-related hyperinsulinemia)	
Subjects	113	71	157	
Age (years)	54 ± 13	61 ± 11*	61 ± 10*	< 0.001
Age on diagnosis (years old)	41 ± 11	44 ± 11*	44 ± 9*	0.005
Sex (male, %)	60 (53.1%)	29 (40.8%)	86 (54.8%)	0.094
Duration of diabetes (years)	13.4 ± 7.1	16.7 ± 9.2*	16.4 ± 7.4*	< 0.001
SBP (mmHg)	132 ± 20	137 ± 20	135 ± 19	0.082
DBP (mmHg)	74 ± 13	75 ± 11	74 ± 10	0.960
BMI (kg/m^2^)	28.9 ± 4.0	27.1 ± 3.2*	26.4 ± 3.2*	< 0.001
FBG (mmol/L)	7.8 ± 2.6	7.8 ± 2.7	7.8 ± 2.8	0.849
HbA1c (mmol/mol)	76.0 ± 6.0	74.9 ± 6.0	76.0 ± 3.8	
HbA1c (%)	9.1 ± 1.6	9.0 ± 1.6	9.1 ± 1.8	0.895
C-peptide (ng/mL)	2.93 ± 1.60	2.45 ± 1.66*	2.01 ± 1.15*^#^	< 0.001
HOMA2-IR HOMA2-B (%)	7.36 ± 3.90 179 (117, 249)	5.96 ± 3.77* 141 (94, 207)	5.06 ± 2.87* 132 (80, 189)*	< 0.001 0.003
Waist circumference (cm) Male Female	101.0 ± 9.3 100.8 ± 9.0	98.3 ± 7.4 94.4 ± 10.6*	97.0 ± 7.7* 93.0 ± 10.4*	0.015 < 0.001
ALT (U/L)	25 (16, 35)	18 (14, 27)*	18 (13, 24)*	< 0.001
AST (U/L)	20 (16, 27)	19 (15, 25)	18 (15, 22)*	0.005
UA (Male, umol/L)	388 ± 98	368 ± 83	377 ± 87	0.559
UA (Female, umol/L)	342 ± 79	298 ± 74*	319 ± 87	0.022
LDL-C (mmol/L)	2.68 ± 0.84	2.66 ± 0.79	2.46 ± 0.81	0.088
TC (mmol/L)	4.33 ± 1.28	4.31 ± 0.99	4.03 ± 1.03	0.086
TG (mmol/L)	1.93 (1.39, 2.83)	1.54 (1.25, 2.39)*	1.46 (1.13, 2.15)*	< 0.001
HDL-C (Male, mmol/L)	0.90 ± 0.19	0.94 ± 0.20	0.97 ± 0.21	0.148
HDL-C (Female, mmol/L)	1.00 ± 0.20	1.13 ± 0.26*	1.11 ± 0.23*	0.007
eGFR (mL/min.1.73m^2^)	103 ± 17	99 ± 13	98 ± 13*	0.024
FINS before PEG (uIU/mL) UACR (mg/g)	20.2 (16.8, 24.5) 13.9 (6.4, 42.6)	42.4 (28.1, 113.9)* 8.7 (5.2, 39.3)	25.0 (18.6,44.9)^#^ 8.7 (4.6, 46.4)	< 0.001 0.057
Diabetic retinopathy, *n* (%)	37 (32.7%)	27 (38.0%)	60 (38.2%)	0.425
Hypertension, *n* (%)	71 (62.8%)	38 (53.5%)	99 (63.1%)	0.309
Hypoglycemia, *n* (%)	39 (34.5%)	30 (42.3%)	75 (47.8%)*	0.028
Obesity, *n* (%)	62 (54.9%)	26 (36.6%)*	42 (26.8%)*	< 0.001
Metabolic syndrome, *n* (%)	102 (90.3%)	52 (73.2%)*	117 (74.5%)*	0.001

BMI, body mass index; SBP, systolic blood pressure; DBP, diastolic blood pressure; MS, metabolism syndromes; LDL-C, low density lipoprotein-cholesterol; TC, total cholesterol; HDL-C, high density lipoprotein-cholesterol; FBG, fasting blood glucose; HbA1c, glycosylated hemoglobin A1c; FINS before PEG, fasting insulin concentration before PEG-precipitation; FINS after PEG, fasting insulin concentration after PEG-precipitation; PPIR, PEG-precipitated insulin ratio; IA, insulin autoimmune antibody; UACR, urinary albumin creatinine rate. * Means p < 0.05 compared to subjects with real hyperinsulinemia. ^#^means p < 0.05 compared to subjects with Real+IAs-related hyperinsulinemia.

### 3.5 IAs may aggravate glucose fluctuation

To further investigate the effects of hyperinsulinemia and its distinct mechanisms on glucose control, glucose fluctuations, and risks of hypoglycemia, FGMs were performed on 22 subjects with IAs that were randomly selected from patient population 1, and on 22 age-, sex-, and HbA1c level-matched subjects without IAs (patient population 2). Compared with subjects without IAs, those with IAs showed a significantly higher CV (32.5% ± 5.3% *vs*. 28.5% ± 5.2%, *p* = 0.024), SD (2.81 ± 0.54 *vs*. 2.34 ± 0.79mmol/L, *p* = 0.045), mean amplitude glycemic differences (MAGE) (6.29 ± 1.06 *vs*. 5.14 ± 1.87mmol/L, *p* = 0.023), and mean of daily differences (MODD) (2.20 ± 0.71 *vs*. 1.60 ± 0.71mmol/L, *p* = 0.004). We found that the mean times below range (TBRs) were higher for subjects with IAs than for those without IAs, although these differences were not statistically significant. There were no statistically significant differences in the duration of diabetes, BMI, mean glucose, serum C-peptide levels, insulin regimens, insulin dosages and duration of insulin therapy between the two groups (**Table 3**).

**Table 3:** Comparisons of glucose parameters and glucose variability metrics between the two groups.

Variables	Group 1	Group 2	*p*-value
Subjects (*n*)	22	22	
Age (years)	63 ± 9	62±11	0.808
Sex (male, %) BMI (kg/m^2^)	9, 40.9% 27.8 ± 1.6	9, 40.9% 25.2 ± 3.6	NA 0.149
HbA1c (mmol/mol)	67.2 ± 9.3	68.3 ± 8.2	
HbA1c (%)	8.3 ± 1.3	8.4 ± 1.4	0.478
C-peptide (ng/mL)	2.11 ± 0.78	2.35 ± 1.17	0.528
FINS (uIU/mL)	28.2 (18.7,45.6)	24.8 (18.3,42.4)	0.410
Duration of diabetes (years)	16.5 ± 5.8	16.9 ± 6.5	0.817
Insulin strategies			
Insulin dosages (IU/d)	40 ± 18	37 ± 22	0.584
Duration of insulin therapy (years)	5.6 ± 4.3	6.9 ± 7.4	0.503
Usage of insulin analog (%)	18 (81.8%)	19 (86.4%)	0.329
Usage of human insulin (%)	9 (40.9%)	10 (45.5%)	0.765
Insulin regimens Rapid, *n* (%) Pre-mixed, *n* (%) Basal bolus, *n* (%) Basal, *n* (%)	0 (0%) 12 (54.5%) 5 (22.7%) 5 (22.7%)	1 (4.5%) 9 (40.9%) 4 (18.2%) 8 (36.4%)	0.643
Mean glucose (mmol/L)	8.78 ± 1.61	8.17 ± 2.09	0.321
TIR (%)	63.2 ± 18.6	73.3 ± 21.0	0.106
TBR (%)	4.2 ± 4.9	3.7 ± 5.2	0.741
TAR (%)	32.6 ± 21.1	23.0 ± 22.7	0.167
CV (%)	32.5 ± 5.3	28.5 ± 5.2	0.024
SD (mmol/L)	2.81 ± 0.54	2.34 ± 0.79	0.045
MAGE (mmol/L)	6.29 ± 1.06	5.14 ± 1.87	0.023
MODD (mmol/L)	2.20 ± 0.71	1.60 ± 0.71	0.004

BMI, body mass index; HbA1c, glycosylated hemoglobin A1c; TIR, time in range; TBR, time below Range; TAR, time above range; CV, coefficient of variability; SD, standard deviation; MAGE, mean amplitude glycemic excursions MODD, mean of daily differences.

### 3.6 FINS/serum C-peptide ratio could help differentiate different types of hyperinsulinemia in clinical practice

**Figure 4:**
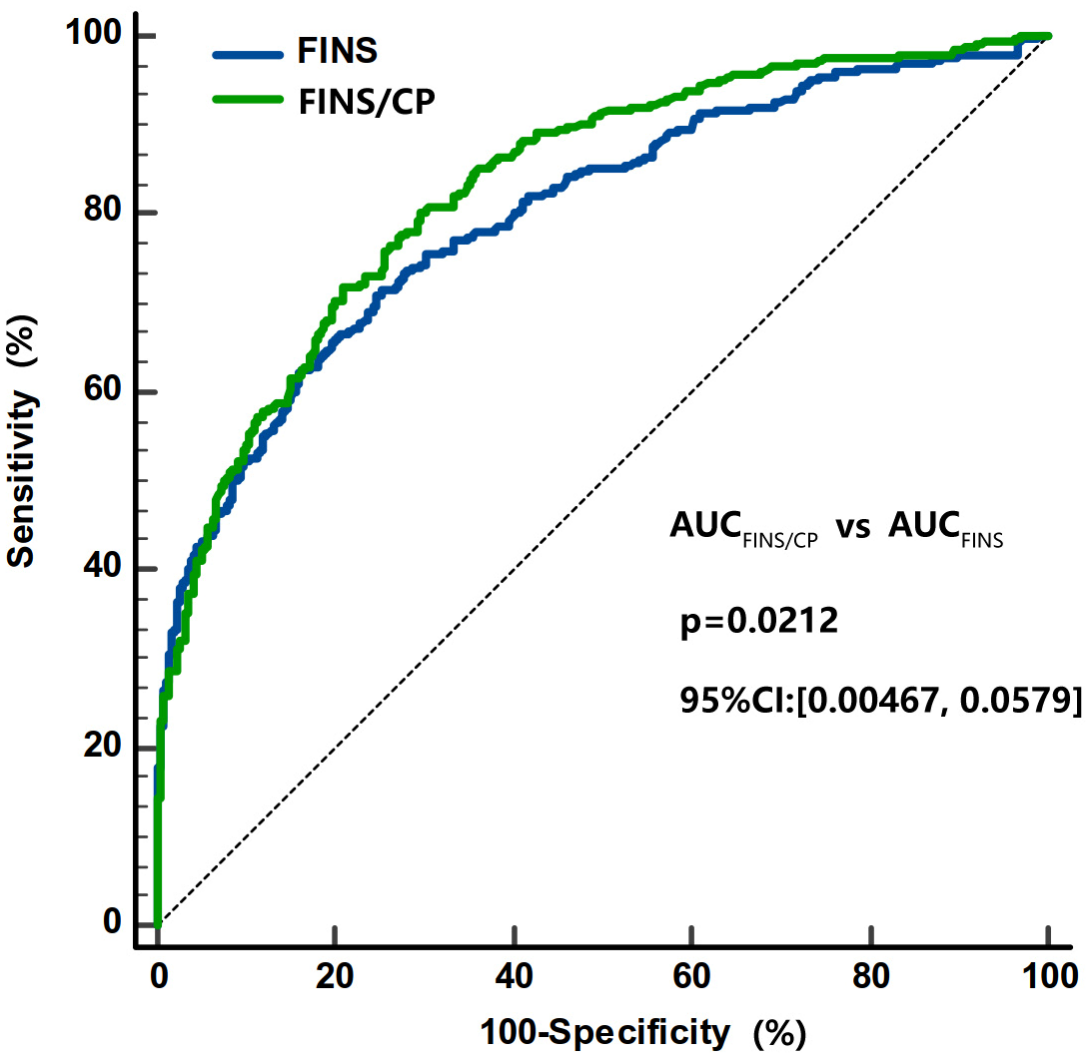
Comparisons of the AUC under ROC curve between FINS/CP and FINS as screening parameters for IAs. The AUC of FINS/CP and FINS for diagnosing IAs is 0.819 (95% CI: 0.776 to 0.863) and 0.764 (95% CI: 0.715 to 0.813), respectively. AUC, area under the curve; FINS, fasting serum insulin; FINS/CP, FINS/serum C-peptide; IAs, insulin antibodies; ROC, receiver operation characteristic.

The FINS/serum C-peptide (FINS/CP) ratio was calculated and was found to be significantly higher in subjects with IAs than in subjects without IAs [17.2 (10.6, 37.5) *vs*. 7.9 (5.6, 11.6), *p* < 0.001]. The AUC for the FINS/CP ratio was 0.819 (95% CI: 0.776, 0.863) and greater than AUC for FINS [0.764 (0.715, 0.813)] for predicting IAs (*p* < 0.001, **Figure 4**). The cutoff for the FINS/CP ratio was 9.3μIU/ng (0.19 molar ratio) for predicting IAs with a sensitivity of 83.3% and a specificity of 70.0%. If we increased the sensitivity to 90.0%, to identify as many IAs-positive individuals as possible in clinical practice, the cutoff for the FINS/CP ratio became 7.5μIU/ng (0.15 molar ratio).

## 4 Discussion

In this study, we found that 341 out of 779 (43.8%) subjects with insulin-treated type 2 diabetes presented with hyperinsulinemia, of whom 113 subjects (33.1%) had real hyperinsulinemia, and the remaining subjects (66.9%) were IAs-positive. There were great differences in insulin resistance and components of MS or beta-cell function between patients with IAs-related hyperinsulinemia and real hyperinsulinemia; the former group had worse beta-cell function, and the latter was more insulin resistant. In addition, the existence of IAs was associated with an increased risk of hypoglycemia and glucose variability. A FINS/CP ratio of over 9.3uIU/ng (0.19 molar ratio) was shown to be a good indicator for screening IAs positive in insulin or insulin analog-treated patients with hyperinsulinemia in clinical practice. For such patients, measurements of FINS levels, and, subsequently, PEG precipitation could help clinicians to recognize hyperinsulinemia with two distinct mechanisms and further tailor suitable therapy strategies.

It is crucial to establish accurate and practical assays of IAs. RIAs with high sensitivity and specificity are particularly promising tools for the detection of IAs (11). However, assay incubation with RIAs takes a long time and requires the disposal of radioactive products, which limits its clinical utility. Commercial electrochemiluminescence kits for IAs target only one-class immunoglobins, such as immunoglobin G (IgG), and showed false-negative results if other insulin antibodies of different classes, such as IgM or IgA, were dominant. Despite their wide availability, the performance of different ELISA kits, as seen in present study, was quite poor (11). It is known that PEG precipitation is a simple, accessible, and inexpensive method that is capable of detecting all types of IAs, and the remarkable difference in FINS before and after PEG precipitation has often been used to determine the presence of IAs in other case reports (12). However, PEG precipitation has seldom been applied in routine clinical practice and the diagnostic criteria based on the PEG precipitation for IAs are not well established. One previous study from Japan established the cutoff for PPIR values for detecting IAs based on control subjects (13). However, that reference range was limited because it was derived from a small sample size of 23 control subjects, and not a large populaiton with diabetes and its effectiveness has not been validated through comparing the performance between their method and RIA. In our study, the established PPIR cutoff (≥10.2%) for predicting IAs is very close to the 97.5th percentile of PPIR in subjects with N-INS(9.9%), showed comparable sensitivity and specificity with RIAs, and showed a higher sensitivity than other ELISA kits. Thus, the calculated PPIR cutoff based on PEG precipitation has important implications for testing in clinical laboratories, allowing for high throughout-testing that is less technically challenging and avoiding the use of radioactive substances.

We found that the incidence of hypoglycemia in patients with hyperinsulinemia and positive IAs was higher than in those with hyperinsulinemia and negative IAs. IAs could prevent insulin from binding its receptor, possibly resulting in the deterioration of the physiological effects of insulin; thereafter, insulin is released from the complexes irrespective of blood glucose concentrations, thus inducing hypoglycemia (14–18). However, the relationship between IAs and hypoglycemia was not determined in previous large-scale studies (13, 19, 20). One of the most important reasons for discrepancy among these findings may be the uncertainty of collecting information related to mild hypoglycemia based on subjects’ retrospective memory. Nevertheless, few studies have focused on the effects of IAs in terms of their role in the fluctuation of blood glucose levels, which are known to play an important role in the development of diabetic complications and affect patients with diabetes’ quality of life (21). We found convincing evidence that IAs increased both within-day and between-day fluctuations in blood glucose levels, as indicated by higher values of CV, SD, MAGE, and MODD in subjects with IAs. The trend of increased risk of hypoglycemia in subjects with IAs-related hyperinsulinemia was also observed, although this was not statistically significant. It has been found that greater fluctuations in blood glucose levels can increase the risk of diabetic complications, such as retinopathy, cardiovascular disease, and mortality, even in patients with the same HbA1c levels (22, 23). Therefore, continuous-use insulin regimens that produce IAs could be less beneficial and replacement with alternative agents may relieve IAs-related fluctuation in blood glucose levels. Certainly, a future study with a larger sample size is needed to confirm our findings.

Two forms of hyperinsulinemia with different mechanisms were identified through PEG precipitation in type 2 diabetic patients who were receiving insulin or insulin analog treatments. In subjects with real hyperinsulinemia, we observed insulin resistance, including high levels of triglyceride, central obesity, and high levels of HOMA2-IR, whereas, among subjects with IAs, 31.1% of subjects still had hyperinsulinemia after PEG precipitation and showed some manifestations of insulin resistance. On the contrary, the subjects with positive IAs did not present with hyperinsulinemia again after PEG precipitation and showed worse beta-cell function. Thus, classifying the forms of hyperinsulinemia has important implications for therapeutic strategy decisions. Theoretically, then, it is preferable for subjects with real hyperinsulinemia to quit or simplify insulin regimens and choose hypoglycemic agents that can improve insulin resistance, such as metformin, sodium-glucose cotransporter 2 (SGLT2) inhibitors, or glucagon-like peptide 1 receptor agonist (GLP-1RAs). In contrast, for subjects with IAs-related hyperinsulinemia, who often have worse beta-cell function and a high risk of hypoglycemia, a combination with SGLT2i should be monitored frequently, since diabetic ketoacidosis (DKA) tends to occur in patients with insulin-deficient diabetes, and SGLT2 inhibitors-associated DKA was reported previously (24).

**Figure 5:**
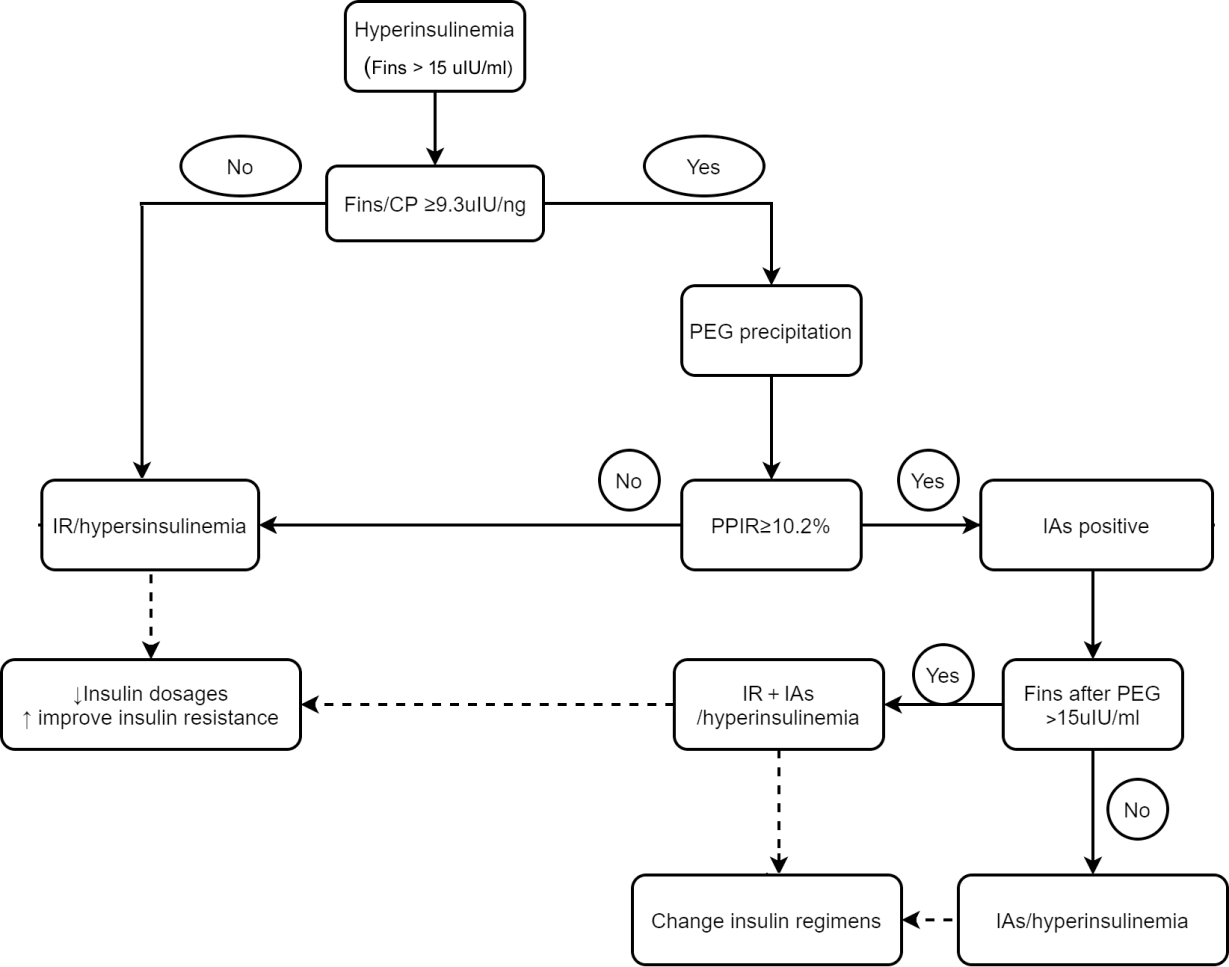
The schematic diagram for differentiating hyperinsulinemia with distinct mechanisms and directing clinical-decision making. IR, insulin resistant; IA, insulin antibody.

Although PEG precipitation is an accurate and convenient assay for IAs measurement, it would be more cost effective to perform PEG precipitation in highly suspected patients rather than in all patients receiving insulin or insulin analogs treatment. Patients with IAs usually have a higher FINS level, an unaffected C-peptide concentration and a higher FINS/CP ratio (25–27).We found that the FINS/CP ratio ≥ 9.3uIU/ng (0.19 molar ratio) demonstrated high sensitivity for screening the presence of IAs in patients with C-INS and hyperinsulinemia. Once hyperinsulinemia is confirmed by detecting FINS in a patient with insulin or insulin analog-treated type 2 diabetes, the FINS/CP ratio should be calculated and, subsequently, a more precise assay such as RIA, or PEG precipitation, carried out. In some cases, although the presence of IAs was verified, hyperinsulinemia remained after PEG precipitation. This suggests that both real hyperinsulinemia and IAs-related hyperinsulinemia were present simultaneously in one patient (**Figure 5**).

Our study has some limitations. First, it is a single-center study. This means that there is a potential selection bias in the study subjects that might limit the representativeness of our findings and their generalizability to other people with type 2 diabetes receiving insulin therapy. Second, the information on hypoglycemia might not be accurate due to the retrospective nature of information collection, and the fact that not all episodes of hypoglycemia were confirmed by glucose measurement. Last, although the kit we used to measure FINS in this study has shown no cross-reactivity with insulin aspart, insulin lispro, and insulin glargine, all of which were widely used by the subjects in our study, a small number of patients used insulin detemir, and the cross-reactivity data for this analog are still undocumented. However, the insulin detemir users accounted for only 3.1% of insulin users and the PPIR-predicted IAs was not affected since the same assays were performed for FINS levels measurement before and after PEG precipitation.

In conclusion, routinely measuring FINS levels was necessary in subjects with type 2 diabetes who were receiving insulin or insulin analogs treatment. The determination of FINS/CP ratios and subsequent PEG precipitation should also be performed to differentiate real hyperinsulinemia from IAs-related hyperinsulinemia, which has different clinical characteristics and pathogenesis. Our findings would provide new strategies for the appropriate and individualized application of hypoglycemic agents in patients with type 2 diabetes.

## Data availability statement

The raw data supporting the conclusions of this article will be made available by the authors, without undue reservation.

## Ethics statement

The studies involving human subjects were reviewed and approved by the Ethics Committee of Peking University People’s Hospital. The patients/subjects provided their written informed consent to participate in this study.

## Author contributions

LZ and YL performed the experiment, data analyses, and wrote the manuscript, YW and YC performed the experiment, RZ, SZ, and SG contributed to collect data, XH and JL proposed the conception of the study and revised the manuscript. All authors contributed to the article and approved the submitted version.
